# Optimizing airway wall segmentation and quantification by reducing the influence of adjacent vessels and intravascular contrast material with a modified integral-based algorithm in quantitative computed tomography

**DOI:** 10.1371/journal.pone.0237939

**Published:** 2020-08-19

**Authors:** Philip Konietzke, Oliver Weinheimer, Willi L. Wagner, Felix Wuennemann, Christian Hintze, Juergen Biederer, Claus P. Heussel, Hans-Ulrich Kauczor, Mark O. Wielpütz

**Affiliations:** 1 Department of Diagnostic and Interventional Radiology, University Hospital of Heidelberg, Heidelberg, Germany; 2 Translational Lung Research Center Heidelberg (TLRC), German Center for Lung Research (DZL), Heidelberg, Germany; 3 Department of Diagnostic and Interventional Radiology with Nuclear Medicine, Thoraxklinik at University of Heidelberg, Heidelberg, Germany; 4 Department of Diagnostic Radiology, University Hospital Schleswig-Holstein, Kiel, Germany; 5 Department of Radiology, German Cancer Research Center (DKFZ), Heidelberg, Germany; 6 Radiologie Rein-Nahe, Bingen, Germany; Clinic for Infectious and tropical diseases, Clinical centre of Serbia, SERBIA

## Abstract

**Introduction:**

Quantitative analysis of multi-detector computed tomography (MDCT) plays an increasingly important role in assessing airway disease. Depending on the algorithms used, airway dimensions may be over- or underestimated, primarily if contrast material was used. Therefore, we tested a modified integral-based method (IBM) to address this problem.

**Methods:**

Temporally resolved cine-MDCT was performed in seven ventilated pigs in breath-hold during iodinated contrast material (CM) infusion over 60s. Identical slices in non-enhanced (NE), pulmonary-arterial (PA), systemic-arterial (SA), and venous phase (VE) were subjected to an in-house software using a standard and a modified IBM. Total diameter (TD), lumen area (LA), wall area (WA), and wall thickness (WT) were measured for ten extra- and six intrapulmonary airways.

**Results:**

The modified IBM significantly reduced TD by 7.6%, LA by 12.7%, WA by 9.7%, and WT by 3.9% compared to standard IBM on non-enhanced CT (p<0.05). Using standard IBM, CM led to a decrease of all airway parameters compared to NE. For example, LA decreased from 80.85±49.26mm^2^ at NE, to 75.14±47.96mm^2^ (-7.1%) at PA (p<0.001), 74.96±48.55mm^2^ (-7.3%) at SA (p<0.001), and to 78.95±48.94mm^2^ (-2.4%) at VE (p = 0.200). Using modified IBM, the differences were reduced to -3.1% at PA, -2.9% at SA and -0.7% at VE (p<0.001; p<0.001; p = 1.000).

**Conclusions:**

The modified IBM can optimize airway wall segmentation and reduce the influence of CM on quantitative CT. This allows a more precise measurement as well as potentially the comparison of enhanced with non-enhanced scans in inflammatory airway disease.

## Introduction

Multi-detector computed tomography (MDCT) is the standard modality for airway imaging [1–3]. Quantitative airway analysis has drawn increasing interest as software tools allow the quantification of airway dimensions of the whole tracheobronchial tree based on thin-section MDCT. Thereby investigators may objectively measure parameters such as lumen area (LA), inner and outer airway diameters, wall thickness (WT), wall area (WA), wall attenuation, airway segment lengths, airway taper indices, and airway branching patterns [1, 2, 4–7]. Bronchial wall remodeling was histopathologically described in lung diseases like asthma and COPD [8], showing corresponding changes in bronchial dimensions on MDCT such as airway dilatation and wall-thickening [9–12]. CT measurements are consistently accurate and reproducible in airway diameters down to approximately 2 mm [3, 13], meaning that intrapulmonary airways of higher generations are below the resolution limit. However, Nakano et al. showed that CT measurements of airways with a Pi of 0.75 cm or more could be used to estimate the dimensions of the small conducting airways [14]. Some parameters measured on MDCT may also correlate with regional lung function [15–18] and might be useful to monitor therapy response [19]. However, a consensus on which parameters to measure in which airway disease is pending [20, 21].

A common method for measurement is based on the full-width-at-half-maximum (FWHM) principle [22]. However, this method may overestimate wall thickness (WT) [23], and subsequently, improved algorithms such as the integral-based method (IBM) were developed to address this problem [6, 24–28]. Iodinated contrast material significantly alters the results of lung density-based quantification of emphysema [29]. Therefore, non-enhanced scans are usually required for quantitative CT in airway disease [1, 2], since contrast material in the vasculature adjacent to intrapulmonary airways might also influence measurements of airway dimensions [7]. Contrast uptake of the airway wall may indicate active inflammation, as opposed to wall remodeling or luminal mucus obstruction. Thus contrast-enhanced studies may be of interest in airway disease.

Therefore, we analyzed the influence of contrast material on airway measurements in different contrast enhancement phases and to test a modified IBM, which can potentially reduce the confounding effects of contrast material on airway dimension measurements. For this purpose, we employed temporally resolved cine-MDCT during intravenous contrast injection in an *in vivo* porcine model, and analyzed airways dimensions using semiautomatic airway analysis using dedicated in-house software.

## Materials and methods

### Animal preparation

All animal studies have been approved by the ethics committee of the Ministry of Agriculture, Environment and, Rural Areas in Kiel, Germany, and were performed in accordance with federal animal protection regulations. Animals were intubated in supine position, and total intravenous anesthesia was maintained throughout the experiment with the respiration being dependent solely on the mechanical ventilator. Central venous catheterization was performed with the catheter being placed in the superior vena cava. Physiologic parameters, such as blood pressure and heart rate, were monitored continuously. Seven healthy, mature, female domestic pigs (Hohenschulen Experimental Farm, Achterwehr, Germany) with a mean weight of 43 kg (range 40–45 kg) were scanned. The animals underwent an extensive imaging campaign, including SPECT [30]. After the study, the animals were euthanized.

#### CT acquisition

MDCT (SOMATOM Sensation 64, Siemens Healthcare GmbH, Forchheim, Germany) was performed to identify the precise scan level (120 kV, 100 mAs, 1 mm recon slice thickness) after stopping mechanical ventilation. Three-dimensional time-resolved cine-MDCT was initiated (0.3 s^-1^, pitch 0, 120 kV, 100 mAs, collimation 12 x 2.4 mm) at a level just below the carina over a period of 40 s. No dose modulation was used. After a short (3 second) pre-contrast phase, phase-contrast material (Imeron 400, Bracco Imaging SPA, Italy) was administered with dose adjusted to bodyweight (0.75ml/kg) via the central venous catheter at an injection rate of 5 ml/s followed by a 20 ml 0.9% NaCl chaser. The mean total administered contrast material was 32.6 ml, which equals to an iodine dose of 300 mg/kg body weight. A time-resolved three-dimensional stack of twelve slices each 2.4 mm thick was reconstructed using a soft kernel (B10f).

#### Semiautomatic post-processing

**Fig 1:**
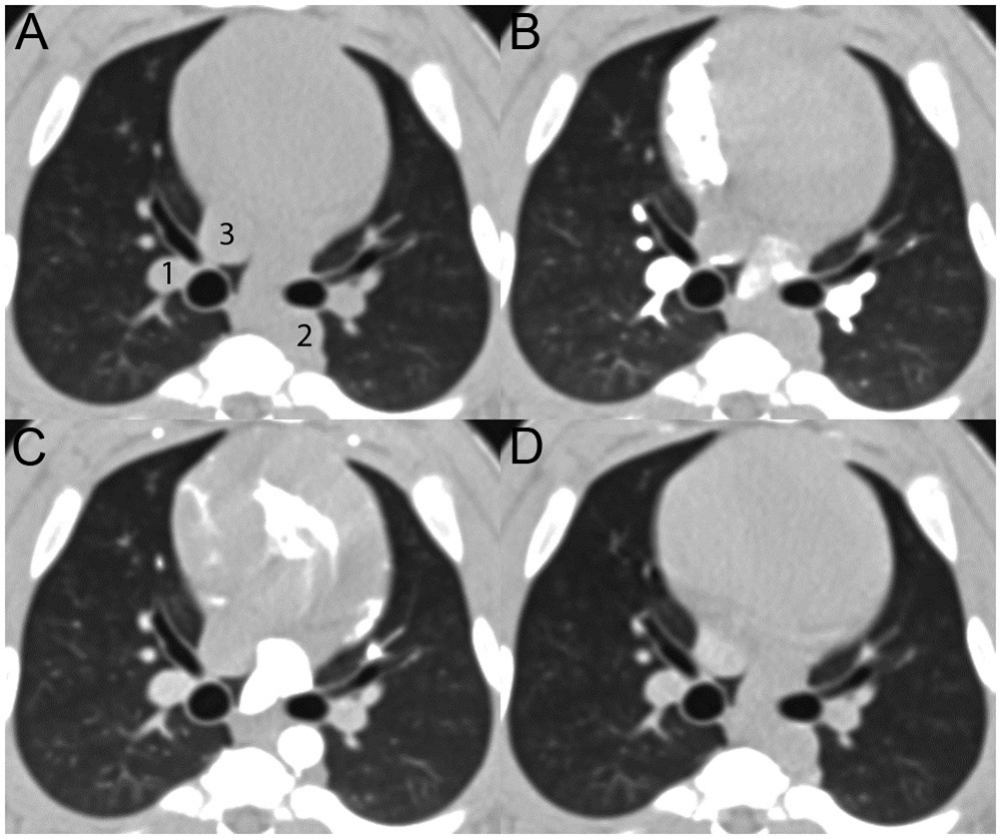
Contrast enhancement phases in cine-MDCT. A–D show the identical slice position at the level of the right intermediate bronchus before (A) and during contrast material perfusion at the time-point of maximum enhancement in the pulmonary artery (B), in the descending aorta (C), and the inferior vena cava (D). 1 indicates the pulmonary artery, 2 the descending aorta, and 3 the inferior cava. Note the density changes of the wall segment of the right intermediate bronchus closely attached to the pulmonary artery, which is due to blurring and smearing of the vascular contrast enhancement into the airway wall.

The in-house software YACTA (“yet another CT analyzer”), a non-commercial scientific software, was employed as described previously [6, 28, 31–35]. The measurements were carried out semiautomatically using the standard IBM algorithm [28] and a modified IBM. Total diameter (TD), lumen area (LA), wall area (WA) and wall thickness (WT) of extrapulmonary main/lobar and intrapulmonary segmental airways, running in close contact with an adjacent artery and almost perpendicular to the axial image plane, were repeatedly measured on identical single slice positions. To determine maximum contrast enhancement in each phase, a 1cm^2^ circular region of interest (ROI) was placed in the right main pulmonary artery, descending aorta and inferior vena cava, and mean density in Hounsfield units (HU) was recorded (Fig 1). Special care was taken to choose images not affected by cardiac pulsation artifacts. Airway dimensions were assessed on four consecutive non-enhanced reconstructions, acquired in the pre-contrast phase of the same three-dimensional dataset, and then each in the pulmonary-arterial phase (PA), in the systemic-arterial phase (SA) and venous phase (VE), based on the time point of maximum enhancement in the respective vascular system. All measurements carried out by a certified chest radiologist with more than 11 years of experience (MOW). A total of n = 10 extrapulmonary (main or lobar bronchi) and n = 6 intrapulmonary segmental bronchi met the requirements for analysis, as described above.

#### Modified integral-based method

**Fig 2:**
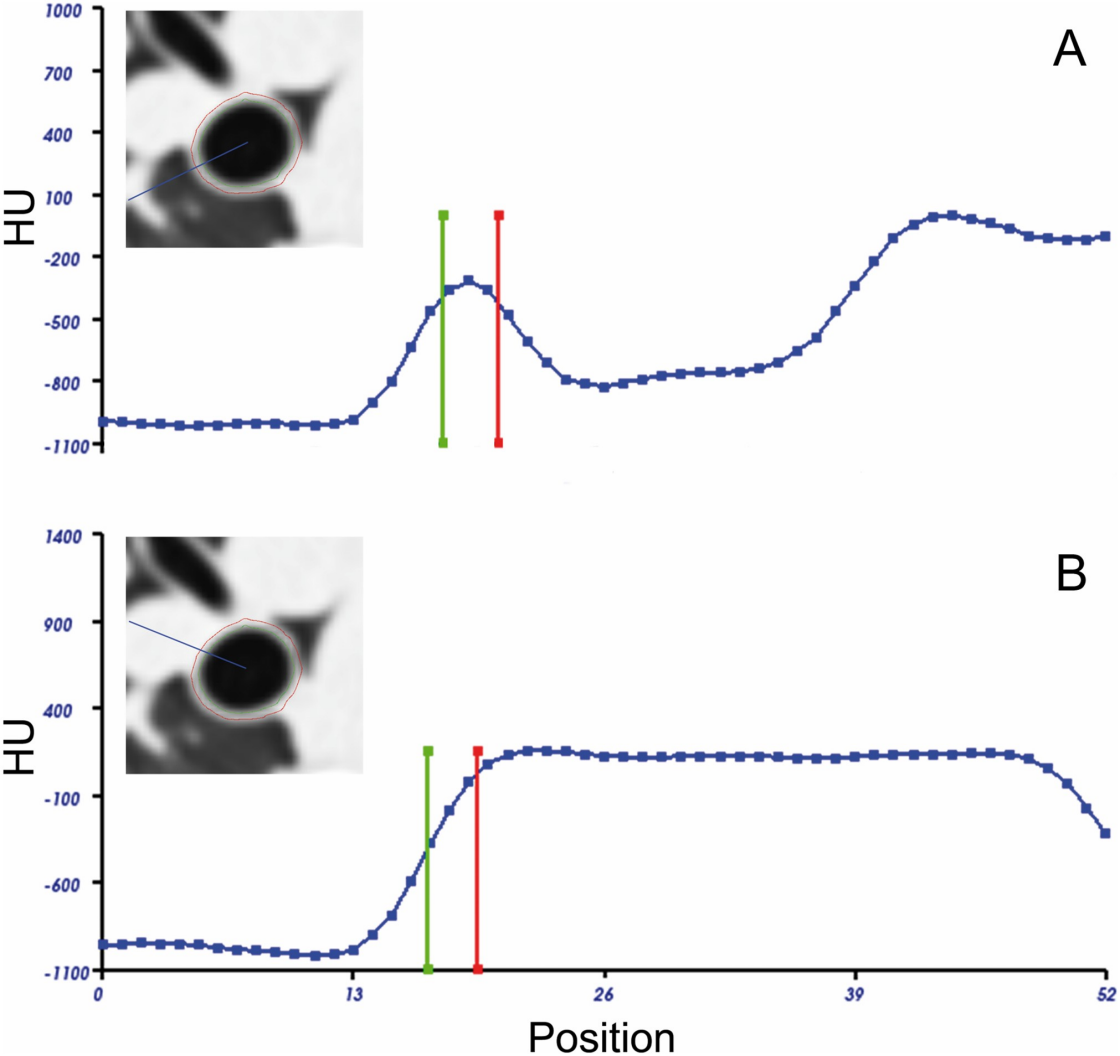
Modified airway wall detection close to vessels. Density profiles across the airway wall in case of surrounding lung parenchyma (non-enhanced) (A) and with an attached pulmonary vessel (B), each as indicated by the radial line superimposed on the computed tomography (CT) image. The detected position of the inner airway wall is indicated as a vertical green, and the outer airway wall border a red vertical bar. Standard lumen detection can fail close to vessels. After modifications to the algorithm, the luminal border near vessels is set when the density profile first reaches the median density as determined from all other valid measurement points (B). Density is given in Hounsfield units (HU).

After the automatic segmentation of the airway tree and computing a centerline, the IBM subsequently recognizes the inner and outer border of an airway wall by calculating the integral value of a density profile along a perpendicular trajectory radiating from the airway center. 128 of such density profiles are computed. The parameters of an ideal airway model are changed so that the integral value of a profile across the model fits the integral value of the profile across the real airway (Fig 2). Caused by low contrast between the wall and the surrounding tissue, the IBM can fail, i.e., in case of an adjacent vessel irrespective of the presence of contrast material. Hence, there is a lack of inner and outer airway wall points occasionally on such profiles. In the standard IBM implementation, the luminal area is calculated by fitting an ellipse to the set of the inner airway wall points in order to compensate for the missing inner airway wall points. At this point, modifications to the IBM were made to the lumen calculation algorithm, whereas the wall thickness measurement algorithm has not been changed: Now, the median HU of all detectable inner airway wall points is determined. Then, on profiles with missing inner airway wall points, these points are defined by the first position on the profile (starting from the lumen center outwards), where this previously determined median HU is located (Fig 2B). Subsequent calculations of airway parameters are the same for standard and modified IBM: All inner airway wall points together are used as polygon vertices, and lumen area (LA) is approximated by the polygon area. Subsequently, the total diameter (TD) was computed as the average distance from the outer to the outer border of an airway segment. Wall thickness (WT) is the median distance between inner and outer border, and wall area (WA) reflects the area within these borders.

#### Statistical analysis

All data were recorded in a dedicated database (Excel, Microsoft Corp., Redmond, USA), and analyses were performed with SigmaPlot (Systat Software GmbH, Erkrath, Germany). The mean and standard deviation (SD) of TD, LA, WA, and WT were calculated separately for each of the four non-enhanced phases as well as for the three contrast phases for lobar and segmental airways and were subsequently pooled for a combined analysis. The results of each of the four consecutive non-enhanced measurements were compared using one-way analysis of variances (ANOVA) for repeated measures, and post-hoc tests with Bonferroni’s correction or Dunn’s method as appropriate in case of multiple comparisons. The results of the four non-enhanced measurements (NE) were considered at baseline and averaged and compared against different contrast phases using the same statistical approach. Statistical differences between standard and modified IBM were compared by paired t-test or Wilcoxon signed-rank test as appropriate. A p-value < 0.05 was considered statistically significant.

## Results

### Reproducibility of airway dimensions on non-enhanced CT

The four non-enhanced, consecutive reconstructions showed good reproducibility without any significant differences being revealed for TD, LA, WA and WT between the four images using either standard or modified IBM (Table 1).

**Table 1:** Reproducibility of airway dimensions on non-enhanced CT.

	**Standard IBM**				
	**NE1**	**NE2**	**NE3**	**NE4**	**p**
**TD [mm]**	11.48±3.89	11.49±3.83	11.47±3.85	11.44±3.87	0.766
**LA [mm**^**2**^**]**	81.01±49.45	80.77±49.12	80.64±49.25	80.97±49.31	0.930
**WA [mm**^**2**^**]**	34.31±17.11	34.50±16.99	34.26±17.13	33.59±16.42	0.378
**WT [mm]**	0.98±0.25	0.99±0.24	0.98±0.26	0.96±0.23	0.251
	**Modified IBM**				
**TD [mm]**	10.58±4.10	10.61±4.07	10.59±4.06	10.63±4.11	0.561
**LA [mm**^**2**^**]**	70.68±47.62	70.35±47.05	69.94±47.05	71.25±47.67	0.608
**WA [mm**^**2**^**]**	30.43±16.86	31.09±17.21	31.12±16.98	30.76±16.90	0.970
**WT [mm]**	0.93±0.24	0.95±0.25	0.96±0.25	0.93±0.24	0.792

Total diameter (TD), lumen area (LA), wall area (WA) and wall-thickness (WT) for combined airways are given as mean ± SD for four consecutive non-enhanced scans. Results were compared with ANOVA on ranks for standard and modified IBM, not showing any significance.

#### Influence of modified IBM on airway dimensions on non-enhanced CT

**Fig 3:**
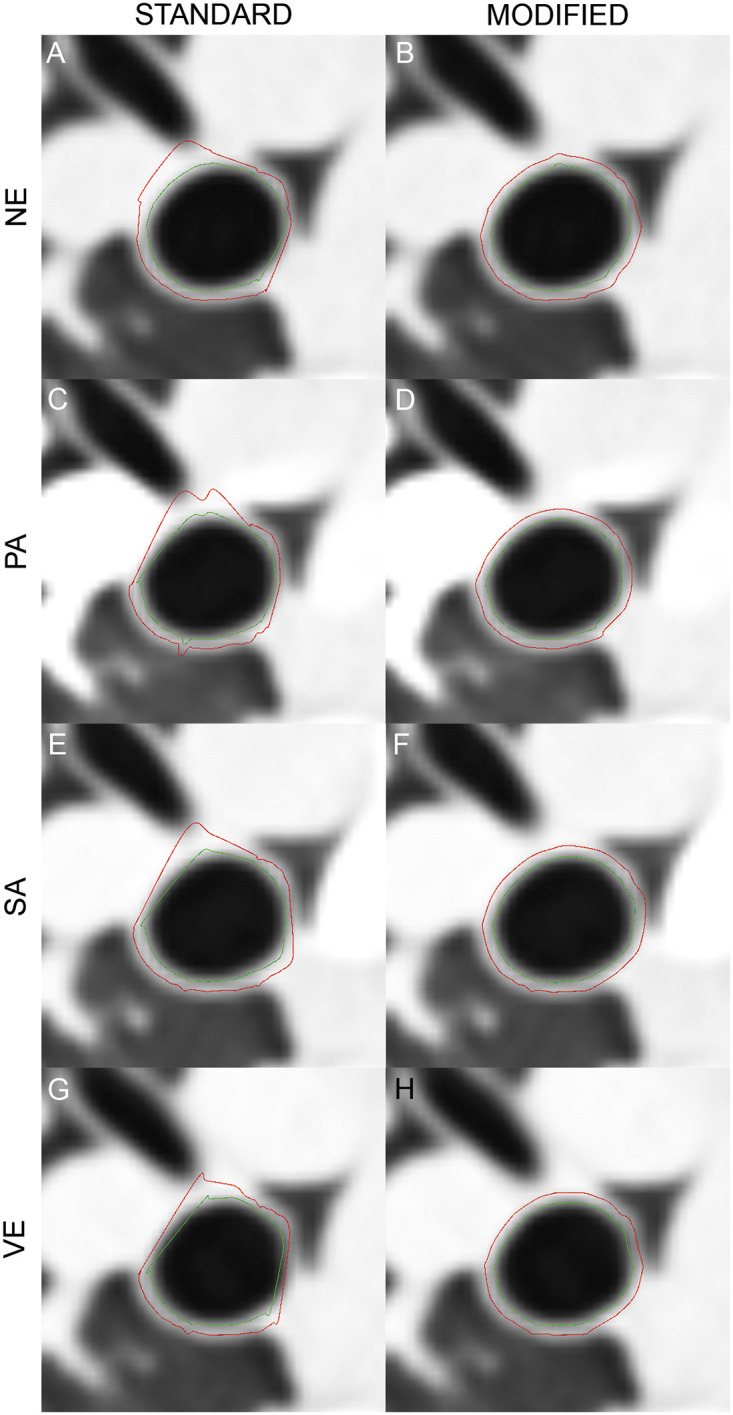
Airway wall detection after contrast material administration. A–H show the results of wall detection of the right intermediate bronchus in non-enhanced images (NE) (A,B), as well as in the pulmonary arterial (PA) (C,D), systemic-arterial (SA) (E,F), and the venous phase (VE) (G,H). Outer (red line) and inner circumference (green line) of the airway wall as calculated by the software are indicated. With the standard algorithm it is evident, that the high intravascular contrast leads to a strong influence on the position calculation of the inner and outer airway border, which is in contact with the vessel (C,E,G). After modification, the wall detection is almost unchanged after contrast administration (D,F,H).

The results of the modification is visualized on Fig 3. Examples of the airway wall detection of the right intermediate bronchus for each contrast phase are shown. The detected margins of the wall segment adjacent to the pulmonary vessel are virtually shifted towards the airway lumen in the contrast enhanced images because of too many missing data points (Fig 3C, 3E and 3G). The displayed error in airway measurements plays a role wherever airways share a large area of contact with the accompanying vessel. This leads to a systematic mismatch with the true airway wall and variation of the airway lumen. After modification of the IBM, the systematic error is reduced (Fig 3D, 3F and 3H). Airway dimensions with the modified IBM showed a significant decrease of all airway parameters compared to the standard IBM. The highest difference was found for LA with -10.29 mm^2^ (-12.7%) (Table 2). This effect was also significantly larger for intrapulmonary segmental airways than for main extrapulmonary airways (S1 Table).

**Table 2:** Influence of modified IBM on airway dimensions on non-enhanced CT.

	Standard	Modified	Δ	Δ(%)	p
**TD [mm]**	11.47±3.86	10.60±4.08	-0.87	-7.55	**<0.001**
**LA [mm**^**2**^**]**	80.85±49.26	70.56±47.34	-10.29	-12.73	**<0.001**
**WA [mm**^**2**^**]**	34.17±16.85	30.85±16.94	-3.32	-9.70	**<0.001**
**WT [mm]**	0.98±0.24	0.94±0.24	-0.04	-3.89	**0.039**

Total diameter (TD), lumen area (LA), wall area (WA) and wall-thickness (WT) for combined airways are presented as mean ± SD. Furthermore, mean differences are given in absolute values (Δ) and % (Δ%). Results for standard and modified IBM were compared by paired t-test or Wilcoxon singed rank test.

#### Influence of contrast material on airway dimensions

In the non-enhanced phase, the mean maximum vessel attenuation in Hounsfield units (HU) was 32 ± 4 HU, showing no significant differences for all target vessels (right pulmonary artery (RPA), descending aorta (DA), and inferior vena cava (IVC)) (Table 3). After contrast material administration, the highest attenuation of 725 ± 95 HU was found in the RPA determining the pulmonary-arterial (PA) phase, followed by 503 ± 57 HU in the DA during the systemic-arterial (SA) phase. In the venous phase (VE), contrast material was evenly distributed in all target vessels, showing no significant differences with a mean of 96 ± 32 HU (Table 3). These results imply that a sharp bolus formation was achieved by the contrast injection protocol.

**Table 3:** Maximum vessel attenuation in contrast enhancement phases.

	RPA [HU]	DA [HU]	IVC [HU]	p
**NE**	35±4	30±7	29±3	0.135
**PA**	725±95	68±43	37±18	**0.002**
**SA**	148±49	503±57	45±15	**<0.001**
**VE**	105±27	85±33	98±31	0.557

Maximum attenuation in Hounsfield units (HU) of the right pulmonary artery (RPA), descending aorta (DA) and inferior vena cava (IVC) in the non-enhanced (NE), pulmonary-arterial (PA), systemic-arterial (SA) and venous (VE) phase. Averaged data from the four non-enhanced reconstructions were used. Data are given as mean ± SD.

Using non-enhanced scans as baseline, contrast material influenced the results of both IBM by causing an overall underestimation of measured airway dimensions. The extent of changes was phase-dependent, with a significant decrease in the PA and SA phase. Comparing the non-enhanced with the VE phase, this influence was not significant (Table 4). This observation did not substantially change when splitting combined airways into main extrapulmonary (S2 Table) and intrapulmonary segmental airways (S3 Table).

**Table 4:** Influence of contrast material on combined airway analysis.

	**Standard IBM**					**Modified IBM**				
	**Pulmonary-arterial phase (PA)**									
	**NE**	**PA**	**Δ**	**Δ(%)**	**p**	**NE**	**PA**	**Δ**	**Δ(%)**	**p**
**TD [mm]**	11.47±3.86	10.93±3.91	-0.54	-4.66	**<0.001**	10.60±4.08	10.31±4.18	-0.29	-2.73	**0.003**
**LA [mm**^**2**^**]**	80.85±49.26	75.14±47.96	-5.71	-7.06	**<0.001**	70.56±47.34	68.37±47.27	-2.19	-3.09	**<0.001**
**WA [mm**^**2**^**]**	34.17±16.85	30.76±15.81	-3.41	-9.96	**<0.001**	30.85±16.94	28.88±16.37	-1.97	-6.38	**0.029**
**WT [mm]**	0.98±0.24	0.92±0.24	-0.06	-6.30	**0.012**	0.94±0.24	0.90±0.24	-0.04	-4.39	0.089
	**Systemic-arterial phase (SA)**									
	**NE**	**SA**	**Δ**	**Δ(%)**	**p**	**NE**	**SA**	**Δ**	**Δ(%)**	**p**
**TD [mm]**	11.47±3.86	10.92±3.98	-0.55	-4.77	**<0.001**	10.60±4.08	10.35±4.21	-0.25	-2.44	**0.010**
**LA [mm**^**2**^**]**	80.85±49.26	74.96±48.55	-5.89	-7.29	**<0.001**	70.56±47.34	68.48±47.58	-2.08	-2.94	**<0.001**
**WA [mm**^**2**^**]**	34.17±16.85	31.21±16.57	-2.96	-8.66	**0.002**	30.85±16.94	29.52±16.98	-1.33	-4.32	0.303
**WT [mm]**	0.98±0.24	0.93±0.23	-0.05	-5.07	0.068	0.94±0.24	0.91±0.24	-0.03	-2.71	0.089
	**Venous phase (VE)**									
	**NE**	**VE**	**Δ**	**Δ(%)**	**p**	**NE**	**VE**	**Δ**	**Δ(%)**	**p**
**TD [mm]**	11.47±3.86	11.27±3.86	-0.20	-1.69	0.132	10.60±4.08	10.54±4.04	-0.06	-0.62	1.000
**LA [mm**^**2**^**]**	80.85±49.26	78.95±48.94	-1.90	-2.35	0.200	70.56±47.34	69.89±47.04	-0.67	-0.94	1.000
**WA [mm**^**2**^**]**	34.17±16.85	32.62±16.10	-1.55	-4.54	0.286	30.85±16.94	30.16±16.59	-0.69	-2.23	1.000
**WT [mm]**	0.98±0.24	0.95±0.21	-0.03	-2.96	0.777	0.94±0.24	0.93±0.23	-0.01	-1.52	0.089

Total diameter (TD), lumen area (LA), wall area (WA) and wall-thickness (WT) were given as mean ± SD. NE results were considered as baseline and differences between pulmonary-arterial (PA), systemic-arterial (SA) and venous phase (VE) are shown as (Δ) and % (Δ%). Standard and modified results for combined airways were tested with ANOVA on ranks test.

The modified IBM could partially compensate for the contrast material effects since the differences between non-enhanced and enhanced scans were overall smaller. For combined airways using the modified IBM, the difference between NE and PA phase was reduced from 10.0% to 6.3% for the wall area (WA).

There were no significant differences when comparing the PA with the SA phase. In contrast, the comparison of both arterial phases and the VE showed significant increases for the airway parameters total diameter (TD) and wall thickness (WT). The increase was less pronounced when using the modified IBM (S4 Table).

## Discussion

Multi-detector computed tomography is the reference modality for airway imaging, and various software tools allow the quantification of airway dimensions [4–7, 19]. Computational airways measurements are still mainly used in scientific contexts, for example, in chronic obstructive pulmonary disease (COPD) [31, 36], bronchial asthma (BA) [5, 16], or cystic fibrosis (CF) [10]. These measurements are usually based on non-enhanced data, since intravenous iodinated contrast material influences the attenuation values of airways and thus the results of airway measurements. Only a few studies have investigated the influence of contrast material on airways and analysis algorithms [7, 29]. Nevertheless, the possibility of performing quantitative CT with contrast media seems desirable, since chest CT examinations are often performed with contrast material, and an additional acquisition without contrast material, only to measure the airway dimensions seems not reasonable. The possibility of quantitatively analyzing contrast-enhanced CT would increase the potential number of data sets without having to apply more radiation. In the above-mentioned lung diseases, increased bronchial angiogenesis and remodeling of pulmonary vessels leads to an altered bronchial and pulmonary circulation [37]. The contrast uptake of the airway wall may indicate active inflammation, as opposed to fibrotic wall remodeling or luminal mucus obstruction. Therefore, quantifying contrast uptake in airways may allow better monitoring of disease activity. Furthermore, the effects of anti-inflammatory drugs might be quantified.

To the best of our knowledge, this is the first study to use cine-MDCT scanning to determine the influence of contrast material application on computational airway analysis in an *in vivo* porcine model. As a first step, we could show that the measurements are highly reproducible in the non-enhanced phase. This information could not be derived from human datasets as multi-phasic MDCT of the chest is rarely performed in a clinical setting, and the measurements of airway dimensions would be further influenced by different inspiration levels between the scans. In this respect, another great advantage of our approach is that we could evaluate the airways at exactly the same slice position within one individual animal, and also at the point of maximum enhancement of each contrast phase.

Based on this, we used the non-enhanced images as a baseline, demonstrating that contrast material caused a decrease of all measured airway parameters when using standard and modified IBM (Fig 3, Table 4). The intensity of the contrast material effect was phase-dependent, with significant decreases of the airway dimensions in the pulmonary-arterial and systemic-arterial phase, whereas non-significant changes were observed in the venous phase. This observation did not substantially change when splitting combined airways into extrapulmonary main and intrapulmonary segmental airways (S3 and S4 Tables). This might indicate that non-enhanced and venous phase scans might be comparable, which is putatively important for the analysis of contrast uptake in airway wall inflammation. The mean density for all three major vessels (right pulmonary artery (RPA), descending aorta (DA), and inferior vena cava (IVC)) was 32 HU, whereas, in the venous phase, it was around 100 HU (Table 3). Accordingly, it may be assumed that below a threshold of approx. 100 HU contrast-related artificial changes in airway dimension measurements might be negligible using standard and modified IBM. Compared to the standard IBM, the modified IBM seems to partially compensate for contrast media effects, especially in the arterial phases, since the differences between non-enhanced and enhanced scans were less pronounced.

The modifications to the IBM also affected the airway dimensions in the non-enhanced scans. The highest difference was found for LA with a significant difference of -10.29 mm^2^ (-12.73%). The impact was also higher on segmental than on main airways. The reason for this might be the decreasing broncho-arterial ratio towards the lung periphery [38]. In smaller airways, the accompanying vessel is relatively larger compared to the bronchus, leading to a larger shared wall portion and, therefore, to a stronger correction by the modified IBM.

The contrast phases had no significant influence on airway measurements when comparing the pulmonary-arterial and the systemic-arterial phase. In contrast, comparing the pulmonary-arterial and the systemic-arterial phase with the venous phase, a significant increase for total diameter (TD) and lumen area (LA) was found. These observations are in line with data published by Dettmer et al. They used data from multi-phasic scans in patients for follow-up of aortic aneurysms, which shows a significant increase in wall thickness after contrast administration in the systemic-arterial phase compared to the venous phase [39]. However, their approach might be intrinsically hampered by the missing control of lung volumes applying repeated breath-hold acquisitions. Furthermore, the tool used by the authors excluded wall segments with adjacent hyperdense structures from the measurement. Thus, conflicting data may partially result from a different approach to deal with missing measurement points in the airway wall, as described in the methods section. To exclude missing data points from airway wall segments with adjacent vessels may not be a sufficient approach, as the wall area affected will increase due to enhancement smear, resulting in artifactual measurement error.

The standard IBM’s accuracy in wall thickness measurements has been evaluated using an anthropomorphic phantom in a previous study. It showed a maximal mean error of 5% for airways with 0.3–2.5 mm WT and 2.6–9.0 mm in diameter and proved to be superior over other algorithms such as the full-width at half-maximum (FWHM) method in small airways [6, 40]. In a subsequent study, the software has been validated in inflation fixed porcine lung explants against histological measurements of TD and WT, with a mean relative error of 5.6–11.0% for airways between 0.37–1.71 mm WT and 3.17–10.74 mm in diameter [27]. These validation experiments compare well to the range of TD and WT detected in our porcine model.

There are some technical limitations to our study. First, a validated gold standard is missing in the study, as no histological correlation was performed. Even if this would be highly desirable, histological validation studies have also restrictions, since the same slices under comparable lung ventilation status have to be compared for valid results. We believe that our results can demonstrate the benefit of the modified IBM’s even without histological correlation. Secondly, we only performed single-slice measurements of the selected bronchi. This is due to the small volume scanned by cine-MDCT, but this technique was also used in similar publications [39]. Thirdly, due to the high iodine dose and the sharp bolus formation, the density achieved in the pulmonary vasculature was higher than in routine human CT protocols [28]. The intention was to challenge the airway measurement algorithm to demonstrate contrast-related artifacts clearly. For the same reason, a soft kernel was chosen, which will emphasize partial volume effects and a smear of contrast from the pulmonary arteries into the airway wall. Lastly, the results may be specific for the used IBM algorithm, since alternative software tools, may use different algorithms, like the full-width at half-maximum (FWHM) [41] or the Laplacian-Gaussian method [10]. Even if it can be assumed that they will produce similar errors in the critical regions near the vasculature, the results cannot directly be transferred to other applications.

## Conclusions

The modified IBM algorithm can reduce the overestimation of airway dimensions as well as the influence of contrast material on quantitative CT. This may allow for a more precise measurement of airway dimensions as well as the comparison of enhanced venous and non-enhanced CT. This might be especially useful for the analysis of contrast-uptake in inflammatory airway diseases.

## Supporting information

S1 Table Influence of modified IBM on repeated non-enhanced CT for different airway sizes. Total diameter (TD), lumen area (LA), wall area (WA) and wall-thickness (WT) for the combined, extrapulmonary main and intrapulmonary segmental airways are presented as mean ± SD. The standard and modified results are tested with t-test or Wilcoxon singed rank test for statistically significant differences A p-value < 0.05 was considered statistically significant.
(PDF)

S2 Table Influence of contrast material on extrapulmonary lobar airway analysis. Total diameter (TD), lumen area (LA), wall area (WA) and wall-thickness (WT) as given mean ± SD. NE scans were considered as baseline and differences between pulmonary-arterial (PA), systemic-arterial (SA) and venous phase (VE) are shown as Δ and Δ (%). Standard and modified results for extrapulmonary main airways were tested with ANOVA on ranks test. A p-value < 0.05 was considered statistically significant.
(PDF)

S3 Table Influence of contrast material on intrapulmonary segmental airway analysis. Total diameter (TD), lumen area (LA), wall area (WA) and wall-thickness (WT) as mean ± SD. NE images were considered as baseline and differences between pulmonary-arterial (PA), systemic-arterial (SA) and venous phase (VE) are shown as Δ and Δ (%). Standard and modified results for intrapulmonary segmental airways were tested with ANOVA on ranks test. A p-value < 0.05 was considered statistically significant.
(PDF)

S4 Table Influence of contrast phase on combined airway analysis. Total diameter (TD), lumen area (LA), wall area (WA) and wall-thickness (WT) of small airways are given as mean ± SD for the non-enhanced phase. Non-enhanced (NE) images were considered as baseline and differences between pulmonary-arterial (PA), systemic-arterial (SA) and venous phase (VE) are shown as Δ and Δ (%). The standard and modified results of two different YACTA versions are compared. Contrast phases vs. NE are tested with ANOVA on ranks for statistically significant differences. A p-value < 0.05 was considered statistically significant.
(PDF)

S1 File Excel data. (XLSX)
