# Supplementary material for: Optimizing airway wall segmentation and quantification by reducing the influence of adjacent vessels and intravascular contrast material with a modified integral-based algorithm in quantitative computed tomography
Source: PLoS One. 2020 Aug 19;15(8):e0237939. doi: 10.1371/journal.pone.0237939 (PMC7437894; doi:10.1371/journal.pone.0237939)
Supplement: S1 Table — Total diameter (TD), lumen area (LA), wall area (WA) and wall-thickness (WT) for the combined, extrapulmonary main and intrapulmonary segmental airways are presented as mean ± SD. The standard and modified results are tested with t-test or Wilcoxon singed rank test for statistically significant differences A p-value < 0.05 was considered statistically significant. (PDF) [file pone.0237939.s001.pdf]

S1 Table. Influence of modified IBM on repeated non-enhanced CT for different airway sizes.

	Combined airways				
	**Standard IBM**	**Modified IBM**	**Δ**	**Δ(%)**	**p**
**TD [mm]**	11.47±3.86	10.60±4.08	-0.87	-7.55	**<0.001**
**LA [mm²]**	80.85±49.26	70.56±47.34	-10.29	-12.73	**<0.001**
**WA [mm²]**	34.17±16.85	30.85±16.94	-3.32	-9.70	**<0.001**
**WT [mm]**	0.98±0.24	0.94±0.24	-0.04	-3.89	**0.039**
	**Extrapulmonary lobar airways**				
**TD [mm]**	14.33±1.22	13.64±1.32	-0.69	-4.80	**<0.001**
**LA [mm²]**	116.68±20.92	104.81±21.22	-11.87	-10.17	**<0.001**
**WA [mm²]**	45.68±9.00	42.62±8.85	-3.06	-6.70	**0.002**
**WT [mm]**	1.09±0.16	1.07±0.15	-0.02	-1.91	0.275
	**Intrapulmonary segmental airways**				
**TD [mm]**	6.71±0.97	5.55±0.80	-1.16	-17.32	**0.007**
**LA [mm²]**	21.13±6.05	13.47±3.30	-7.66	-36.28	**0.010**
**WA [mm²]**	14.98±5.75	11.24±4.39	-3.74	-24.98	**0.031**
**WT [mm]**	0.79±0.24	0.72±0.20	-0.07	-8.46	0.094
