# Supplementary material for: Optimizing airway wall segmentation and quantification by reducing the influence of adjacent vessels and intravascular contrast material with a modified integral-based algorithm in quantitative computed tomography
Source: PLoS One. 2020 Aug 19;15(8):e0237939. doi: 10.1371/journal.pone.0237939 (PMC7437894; doi:10.1371/journal.pone.0237939)
Supplement: S2 Table — Total diameter (TD), lumen area (LA), wall area (WA) and wall-thickness (WT) as given mean ± SD. NE scans were considered as baseline and differences between pulmonary-arterial (PA), systemic-arterial (SA) and venous phase (VE) are shown as Δ and Δ (%). Standard and modified results for extrapulmonary main airways were tested with ANOVA on ranks test. A p-value < 0.05 was considered statistically significant. (PDF) [file pone.0237939.s002.pdf]

S2 Table. Influence of contrast material on extrapulmonary lobar airway analysis.

	Standard IBM					Modified IBM				
	**Pulmonary-arterial phase (PA)**									
	**NE**	**PA**	**Δ**	**Δ(%)**	**p**	**NE**	**PA**	**Δ**	**Δ(%)**	**p**
**TD [mm]**	14.33±1.22	13.85±1.19	-0.48	-3.29	**<0.001**	13.64±1.32	13.42±1.30	-0.22	-1.58	**0.041**
**LA [mm²]**	116.68±20.92	110.01±20.75	-6.67	-5.72	**<0.001**	104.81±21.22	102.58±21.17	-2.23	-2.12	**0.012**
**WA [mm²]**	45.68±9.00	41.82±7.76	-3.86	-8.45	**0.012**	42.62±8.85	40.24±8.50	-2.38	-5.59	0.159
**WT [mm]**	1.09±0.16	1.04±0.14	-0.06	-5.29	0.152	1.07±0.15	1.03±0.15	-0.04	-4.17	0.313
	**Systemic-arterial phase (SA)**									
	**NE**	**SA**	**Δ**	**Δ(%)**	**p**	**NE**	**SA**	**Δ**	**Δ(%)**	**p**
**TD [mm]**	14.33±1.22	13.90±1.28	-0.43	-3.00	**<0.001**	13.64±1.32	13.48±1.35	-0.16	-1.15	0.255
**LA [mm²]**	116.68±20.92	110.19±21.32	-6.49	-5.56	**<0.001**	104.81±21.22	102.81±21.75	-2.00	-1.91	**0.028**
**WA [mm²]**	45.68±9.00	42.74±8.78	-2.94	-6.43	0.089	42.62±8.85	41.34±8.91	-1.28	-3.00	0.159
**WT [mm]**	1.09±0.16	1.05±0.14	-0.04	-3.66	0.152	1.07±0.15	1.05±0.14	-0.02	-1.93	0.313
	**Venous phase (VE)**									
	**NE**	**VE**	**Δ**	**Δ(%)**	**p**	**NE**	**VE**	**Δ**	**Δ(%)**	**p**
**TD [mm]**	14.33±1.22	14.15±1.22	-0.18	-1.23	0.222	13.64±1.32	13.54±1.36	-0.10	-0.72	1.000
**LA [mm²]**	116.68±20.92	114.61±20.71	-2.07	-1.78	0.509	104.81±21.22	103.76±21.82	-1.05	-1.00	0.714
**WA [mm²]**	45.68±9.00	43.78±8.37	-1.90	-4.15	0.626	42.62±8.85	41.65±8.96	-0.97	-2.27	0.159
**WT [mm]**	1.09±0.16	1.06±0.14	-0.03	-3.13	0.152	1.07±0.15	1.05±0.15	-0.02	-1.70	0.313
