# Supplementary material for: Optimizing airway wall segmentation and quantification by reducing the influence of adjacent vessels and intravascular contrast material with a modified integral-based algorithm in quantitative computed tomography
Source: PLoS One. 2020 Aug 19;15(8):e0237939. doi: 10.1371/journal.pone.0237939 (PMC7437894; doi:10.1371/journal.pone.0237939)
Supplement: S3 Table — Total diameter (TD), lumen area (LA), wall area (WA) and wall-thickness (WT) as mean ± SD. NE images were considered as baseline and differences between pulmonary-arterial (PA), systemic-arterial (SA) and venous phase (VE) are shown as Δ and Δ (%). Standard and modified results for intrapulmonary segmental airways were tested with ANOVA on ranks test. A p-value < 0.05 was considered statistically significant. (PDF) [file pone.0237939.s003.pdf]

S3 Table. Influence of contrast material on intrapulmonary segmental airway analysis.

	**Standard IBM**					**Modified IBM**				
	**Pulmonary-arterial phase (PA)**									
	**NE**	**PA**	**Δ**	**Δ(%)**	**p**	**NE**	**PA**	**Δ**	**Δ(%)**	**p**
**TD [mm]**	6.71±0.97	6.07±0.75	-0.64	-9.54	**0.037**	5.55±0.80	5.13±0.88	-0.42	-7.44	0.088
**LA [mm²]**	21.13±6.05	17.03±3.60	-4.10	-19.42	**0.009**	13.47±3.30	11.35±3.12	-2.12	-15.68	0.071
**WA [mm²]**	14.98±5.75	12.34±4.79	-2.64	-17.62	0.018	11.24±4.39	9.96±4.59	-1.28	-11.39	0.086
**WT [mm]**	0.79±0.24	0.72±0.24	-0.07	-8.63	0.099	0.72±0.20	0.68±0.22	-0.04	-4.95	0.444
	**Systemic-arterial phase (SA)**									
	**NE**	**SA**	**Δ**	**Δ(%)**	**p**	**NE**	**SA**	**Δ**	**Δ(%)**	**p**
**TD [mm]**	6.71±0.97	5.97±0.58	-0.74	-11.07	0.066	5.55±0.80	5.12±0.79	-0.43	-7.70	0.106
**LA [mm²]**	21.13±6.05	16.24±3.29	-4.89	-23.17	**0.034**	13.47±3.30	11.27±3.40	-2.20	-16.29	0.088
**WA [mm²]**	14.98±5.75	11.98±3.58	-3.00	-19.99	**0.043**	11.24±4.39	9.81±3.83	-1.43	-12.71	0.086
**WT [mm]**	0.79±0.24	0.72±0.19	-0.07	-8.31	0.099	0.72±0.20	0.69±0.18	-0.03	-4.63	0.444
	**Venous phase (VE)**									
	**NE**	**VE**	**Δ**	**Δ(%)**	**p**	**NE**	**VE**	**Δ**	**Δ(%)**	**p**
**TD [mm]**	6.71±0.97	6.49±0.79	-0.22	-3.33	0.678	5.55±0.80	5.54±0.71	-0.01	-0.21	1.000
**LA [mm²]**	21.13±6.05	19.53±4.64	-1.60	-7.59	1.000	13.47±3.30	13.44±2.97	-0.03	-0.16	1.000
**WA [mm²]**	14.98±5.75	14.00±4.50	-0.98	-6.51	1.000	11.24±4.39	11.02±3.67	-0.22	-1.94	0.086
**WT [mm]**	0.79±0.24	0.77±0.19	-0.02	-2.57	0.099	0.72±0.20	0.71±0.17	-0.01	-1.09	0.444
