# Supplementary material for: Optimizing airway wall segmentation and quantification by reducing the influence of adjacent vessels and intravascular contrast material with a modified integral-based algorithm in quantitative computed tomography
Source: PLoS One. 2020 Aug 19;15(8):e0237939. doi: 10.1371/journal.pone.0237939 (PMC7437894; doi:10.1371/journal.pone.0237939)
Supplement: S4 Table — Total diameter (TD), lumen area (LA), wall area (WA) and wall-thickness (WT) of small airways are given as mean ± SD for the non-enhanced phase. Non-enhanced (NE) images were considered as baseline and differences between pulmonary-arterial (PA), systemic-arterial (SA) and venous phase (VE) are shown as Δ and Δ (%). The standard and modified results of two different YACTA versions are compared. Contrast phases vs. NE are tested with ANOVA on ranks for statistically significant differences. A p-value < 0.05 was considered statistically significant. (PDF) [file pone.0237939.s004.pdf]

S4 Table. Influence of contrast phase on combined airway analysis.

	**Standard IBM**					**Modified IBM**				
	**Pulmonary-arterial (PA) vs. Systemic-arterial (SA) phase**									
	**PA**	**SA**	**Δ**	**Δ(%)**	**p**	**PA**	**SA**	**Δ**	**Δ(%)**	**p**
**TD [mm]**	10.93±3.91	10.92±3.98	-0.01	-0.11	1.000	10.31±4.18	10.35±4.21	0.04	0.31	1.000
**LA [mm²]**	75.14±47.96	74.96±48.55	-0.18	-0.24	1.000	68.37±47.27	68.48±47.58	0.11	0.16	1.000
**WA [mm²]**	30.76±15.81	31.21±16.57	0.44	1.44	1.000	28.88±16.37	29.52±16.98	0.63	2.19	0.215
**WT [mm]**	0.92±0.24	0.93±0.23	0.01	1.32	0.198	0.90±0.24	0.91±0.24	0.02	1.77	0.364
	**Pulmonary-arterial (PA) vs. Venous phase (VE) phase**									
	**PA**	**VE**	**Δ**	**Δ(%)**	**p**	**PA**	**VE**	**Δ**	**Δ(%)**	**p**
**TD [mm]**	10.93±3.91	11.27±3.86	0.34	3.12	**<0.001**	10.31±4.18	10.54±4.04	0.23	2.17	**0.030**
**LA [mm²]**	75.14±47.96	78.95±48.94	3.81	5.07	**<0.001**	68.37±47.27	69.89±47.04	1.52	2.23	**0.008**
**WA [mm²]**	30.76±15.81	32.62±16.10	1.85	6.02	**0.040**	28.88±16.37	30.16±16.59	1.28	4.43	0.215
**WT [mm]**	0.92±0.24	0.95±0.21	0.03	3.56	0.198	0.90±0.24	0.93±0.23	0.03	3.00	0.364
	**Systemic-arterial (SA) vs. Venous phase (VE) phase**									
	**SA**	**VE**	**Δ**	**Δ(%)**	**p**	**SA**	**VE**	**Δ**	**Δ(%)**	**p**
**TD [mm]**	10.92±3.98	11.27±3.86	0.35	3.12	**<0.001**	10.35±4.21	10.54±4.04	0.19	2.17	**0.075**
**LA [mm²]**	74.96±48.55	78.95±48.94	4.00	5.07	**<0.001**	68.48±47.58	69.89±47.04	1.41	2.23	**0.015**
**WA [mm²]**	31.21±16.57	32.62±16.10	1.41	6.02	0.164	29.52±16.98	30.16±16.59	0.65	4.43	0.215
**WT [mm]**	0.93±0.23	0.95±0.21	0.02	3.56	0.198	0.91±0.24	0.93±0.23	0.01	3.00	0.364
